# Chronic or Subacute Arthritis: The Hot Sand Bath Treatment

**Published:** 1907-04-13

**Authors:** 


### Points in Treatment.

### CHRONIC OR SUBACUTE ARTHRITIS: THE HOT SAND BATH TREATMENT.

The value of local heat in the treatment of subacute or chronic joint affections is undoubted. It is even probable that there is no other therapeutic agent which, used alone, can do so much good in these conditions. For the purposes of the present discussion it will be supposed that the patient under treatment is a man of between 20 and 30, who has had gonococcal synovitis of several joints, but who has recovered except as to one knee which remains painful and stiff and threatens to become fixed by pseudo-ankylosis.

Such patients are common enough, and it is often difficult to know what is the best treatment for the knee. Almost everything may have been tried, and yet the joint remains affected subacutely. The method adopted in many hospitals, and in private houses where electric lighting is laid on, is a radiant heat bath, given once a day as follows:—Over the affected leg a tall cradle is arranged; on the top bar are four 32-candle-power electric lamps, which can be connected by a wire to a wall plug and the electric main. Over all is thrown a blanket; outside this a mackintosh sheet, and outside this again the rest of the bed-clothes, the whole of the coverings outside the cradle being most carefully tucked in all around to prevent the escape of any heat. The lamps give off not only light, but also radiant heat; the result, therefore, is that the air inside the cradle becomes gradually hotter and hotter, until the affected knee is submitted to an intense heat.

### The Rationale of the Treatment.

The rationale of the treatment is as follows:—If a piece of meat were subjected to this degree of heat it would be cooked; the living tissue, however, is able to keep down its own temperature below cooking point. This it does in at least two ways: in the first place, by profuse local perspiration, the evaporation of which keeps on taking up heat; secondly, by the mechanical carrying away of the heated blood that has circulated through the limb; the latter factor alone would lead to a greatly accelerated local circulation, but when fresh blood is constantly being required for the production of more sweat, the total increase in the volume of blood that has to circulate round the affected knee when it is subject to such a radiant heat bath is enormous. It is supposed that the benefit of the treatment is mainly due to this increased local circulation; how this in its turn acts is not known, though presumably the more fresh healthy blood flows round and through some forms of diseased structures, the greater is the likelihood of repair. The good results of the treatment are undoubted; nor is it only in gonococcal arthritis which is tending to become chronic that this form of treatment has been extremely good.

### Its Substitute—The Sand Bath.

Unfortunately in many places, both in towns and in the country, the electric radiant heat bath cannot be resorted to. The question arises whether any substitute is possible. There is no other convenient way of subjecting a part to heat at such extremely high local temperatures, so that all substitutes are inferior to the method described above. Nevertheless there is one old-fashioned remedy which has a similar effect, and which, whilst costing almost nothing, can be used in the poorest house; this is the hot sand bath. Plenty of sand is needed; ordinary roadside sand will do, though sharp silver sand is better. If roadside sand be used, at least enough to fill two of the ordinary large cubical biscuit tins should be collected, and first of all well baked at almost a red heat both to dry it and to destroy the organic matter in it and render it aseptic. When once made clean and dry it may be kept in the biscuit tins and used again and again. The patient, when a sand bath is to be given, lies with a mackintosh under the knee or other part to be treated; by a suitable arrangement the mackintosh can be made to form a sort of trough or well in which the knee lies; the object of this is to prevent the sand from spreading too far laterally in any direction away from the knee. When all is ready, and the sand in the tins is so hot that a hand dipped into it can only just be held there, the tins are brought in from the kitchen, and their contents gently poured all round and over the knee, covering both it and about nine inches of the leg above and below it. The amount of sand that can be borne will depend upon the amount of painfulness to pressure there may be; but if possible the knee should be buried several inches deep in the very hot sand. A mackintosh is put over the top, and over this a blanket to keep the heat in, and the knee is left in the sand bath for 20 minutes, half an hour, or more, as the case may be. The only object, apart from its cheapness, of using sand rather than anything else is, of course, the great length of time that it takes to cool. When the bath is done the patient's leg is gently raised; the greater part of the sand remains upon the lower mackintosh, and can be readily poured back into the tins and got ready for the next application. There is still some left sticking to the patient's skin owing to the latter having become bathed in sweat; a pan of water should be brought under the limb, and with a sponge all this adhering sand can in a moment or two be washed off, leaving the limb and the bed both quite clean. It is a very good time to massage the leg now when it is hyperæmic and supple, if massage is required in the particular case. The relief afforded by this simple method of treatment is sometimes wonderful; the knee, the foot, the elbow, and the wrist are the parts to which it can most easily be applied, but with appropriate arrangements it is applicable to the shoulder, the hip, and even to the abdomen, and cases are constantly occurring in which one is but too glad to employ it.

